# Evaluation of in vivo and in vitro binding property of a novel candidate PET tracer for CSF1R imaging and comparison with two currently-used CSF1R-PET tracers

**DOI:** 10.1186/s41181-025-00345-8

**Published:** 2025-05-13

**Authors:** Xiyan Rui, Yuzhou Ding, Nailian Zhang, Xinran Zhao, Chie Seki, Tomoteru Yamasaki, Masayuki Fujinaga, Ming-Rong Zhang, Jun Qian, Bin Ji, Rong Zhou

**Affiliations:** 1 Department of Nephrology, Yangpu Hospital, School of Medicine, Tongji University, 450 Tengyue Road, Shanghai, 200090 China; 2 Department of Radiopharmacy and Molecular Imaging, School of Pharmacy, Fudan University, 826 Zhangheng Road, Shanghai, 201203 China; 3 Advanced Neuroimaging Center, National Institutes for Quantum Science and Technology, Chiba, Japan; 4 Department of Advanced Nuclear Medicine Sciences, Institute for Quantum Medical Science, National Institutes for Quantum Science and Technology, Chiba, Japan; 5 Department of Clinical and Experimental Neuroimaging, Center for Development of Advanced Medicine for Dementia, National Center for Geriatrics and Gerontology, Obu, Japan; 6 National Key Laboratory of Advanced Drug Formulations for Overcoming Delivery Barriers, Shanghai, China; 7 Key Laboratory of Smart Drug Delivery, Fudan University, Ministry of Education, Shanghai, China; 8 Institute for Small-Molecule Drug Discovery & Development, Quzhou Fudan Institute, Quzhou, China

**Keywords:** Positron emission computed tomography (PET), Autoradiography, Colony-stimulating factor 1 receptor (CSF1R), [^11^C]CPPC, [^11^C]*o*-aminopyridyl alkynyl derivative

## Abstract

**Background:**

Colony-stimulating factor 1 receptor (CSF1R) is a promising imaging biomarker for neuroinflammation and tumor-associated macrophages. However, existing positron emission tomography (PET) tracers for CSF1R imaging often suffer from limited specificity or sensitivity.

**Results:**

We have performed ^11^C-labeled radiosynthesis of compound FJRD (3-((2-amino-5-(1-methyl-1*H*-pyrazol-4-yl)pyridin-3-yl)ethynyl)-*N*-(4-methoxyphenyl)-4-methylbenzamide), which exhibits excellent affinity for CSF1R, and evaluated its in vivo and in vitro binding properties. PET images of [^11^C]FJRD show low brain uptake and specific binding in the living organs, except the kidneys in both normal mice and rats. In vitro autoradiographs demonstrate high levels of specific binding in all investigated organs, including the brain, spleen, liver, kidneys and lungs, when self-blocking was used. The addition of CPPC partially blocked in vitro [^11^C]FJRD binding in these organs, with blocking effects ranging from 9 to 67%. In contrast, the other two CSF1R inhibitors, GW2580 and BLZ945, showed minimal blocking effects, suggesting unignorable off-target binding in these organs. Furthermore, specific binding of [^11^C]CPPC and [^11^C]GW2580 was faint in the mouse organs, with [^11^C]CPPC demonstrating detectable binding only in the spleen.

**Conclusions:**

These results suggest that [^11^C]FJRD is a potential CSF1R-PET tracer for more sensitive detection of CSF1R, compared to [^11^C]CPPC and [^11^C]GW2580. However, the high level off-target binding necessitates further improvements in specificity for CSF1R imaging.

## Background

Colony-stimulating factor 1 receptor (CSF1R) is a tyrosine kinase expressed on macrophages in peripheral tissues and on microglia in the central nervous system (CNS) (1–2). Growing evidence highlights the pivotal roles of microglia and macrophages in driving neuroinflammation and establishing an immunosuppressive tumor microenvironment, both of which contribute to the progression of neurological disorders and tumor development (3–4). Given that the survival and proliferation of microglia and macrophages depend on the CSF1R signaling pathway, several CSF1R inhibitors have been developed as potential therapies for neurological disorders and cancer (Cannarile et al. 2017). These treatments either focus on depleting inflammatory microglia from the brain (Green et al. 2020), or on reprogramming microglia and macrophages from an immunosuppressive to an anti-tumor phenotype (Fujiwara et al. 2021). Therefore, non-invasive detection of microglia and macrophages through CSF1R imaging is crucial for monitoring spatiotemporal changes and enabling early medical intervention at the prodromal stages of these diseases. Positron emission tomography (PET), as a non-invasive imaging modality, is well-suited to serve as a valuable bridge between preclinical and clinical applications (7–8). Several CSF1R-PET tracers, including [^11^C]CPPC and [^11^C]GW2580, have been developed to potentially visualize activated microglia in the brains of rodents, non-human primates, and human subjects with neuroinflammation (Zhou et al. 2021; Altomonte et al. 2023; Mills et al. 2024) [^11^C]CPPC is the first PET trace for CSF1R imaging, and it has successfully captured neuroinflammation in several animal models (10, 12–13) and in human patients with neurodegenerative diseases such as Parkinson’s disease (Mills et al. 2024; Coughlin et al. 2022), although CPPC also showed unignorable affinities for other kinases (Knight et al. 2021) [^11^C]GW2580 demonstrated higher accumulation of radioactive signals than [^11^C]CPPC in mouse models with neuroinflammation and in normal non-human primates (Zhou et al. 2021), suggesting its potential capacity for CSF1R imaging. Other several PET tracers, including [(^11^C]NCGG401 [^11^C]AZ683 and [^11^C] Psa374, have also been developed for CSF1R imaging. However, common problems of these CSF1R tracers include a lack of selectivity for other kinases, weak specific signals, and limited brain permeability (10, 15–16). Recently, researchers reported a series of *o*-aminopyridyl alkynyl derivatives with high affinity for CSF1R (Xie et al. 2020). An et al. developed an ^18^F-labeled *o*‑aminopyridyl alkynyl derivative [^18^F]4 (3-((2-amino-5-(1-methyl-1*H*-pyrazol-4-yl)pyridin-3-yl)ethynyl)-*N*-(3-(2-fluoroethoxy)phenyl)-4-methylbenzamide), for CSR1R imaging and demonstrated a 25% increase in brain uptake in lipopolysaccharide-treated mice compared to normal controls (An et al. 2023). This suggests that [^18^F]4 holds potential as an imaging agent for CSF1R. However, the specificity and superiority of *o*-aminopyridyl alkynyl derivatives as CSF1R-PET tracer remains unclear due to the lack of data on in vitro binding properties and comparison with other CSF1R imaging tracers. To address these concerns, in this study, we conducted the ^11^C-labeled radiosynthesis of FJRD (3-((2-amino-5-(1-methyl-1*H*-pyrazol-4-yl)pyridin-3-yl)ethynyl)-*N*-(4-methoxyphenyl)-4-methylbenzamide), a reported *o*-aminopyridyl alkynyl derivative with high affinity for CSF1R (*IC*_*50*_ = 1.4 nM) (Xie et al. 2020), and performed in vitro and in vivo evaluations in normal rodents. Additionally, we compared its in vitro binding properties with two currently-used CSF1R-PET tracers [^11^C]CPPC and [^11^C]GW2580.

## Results

### Chemical synthesis and radiolabeling

**Fig. 1:**
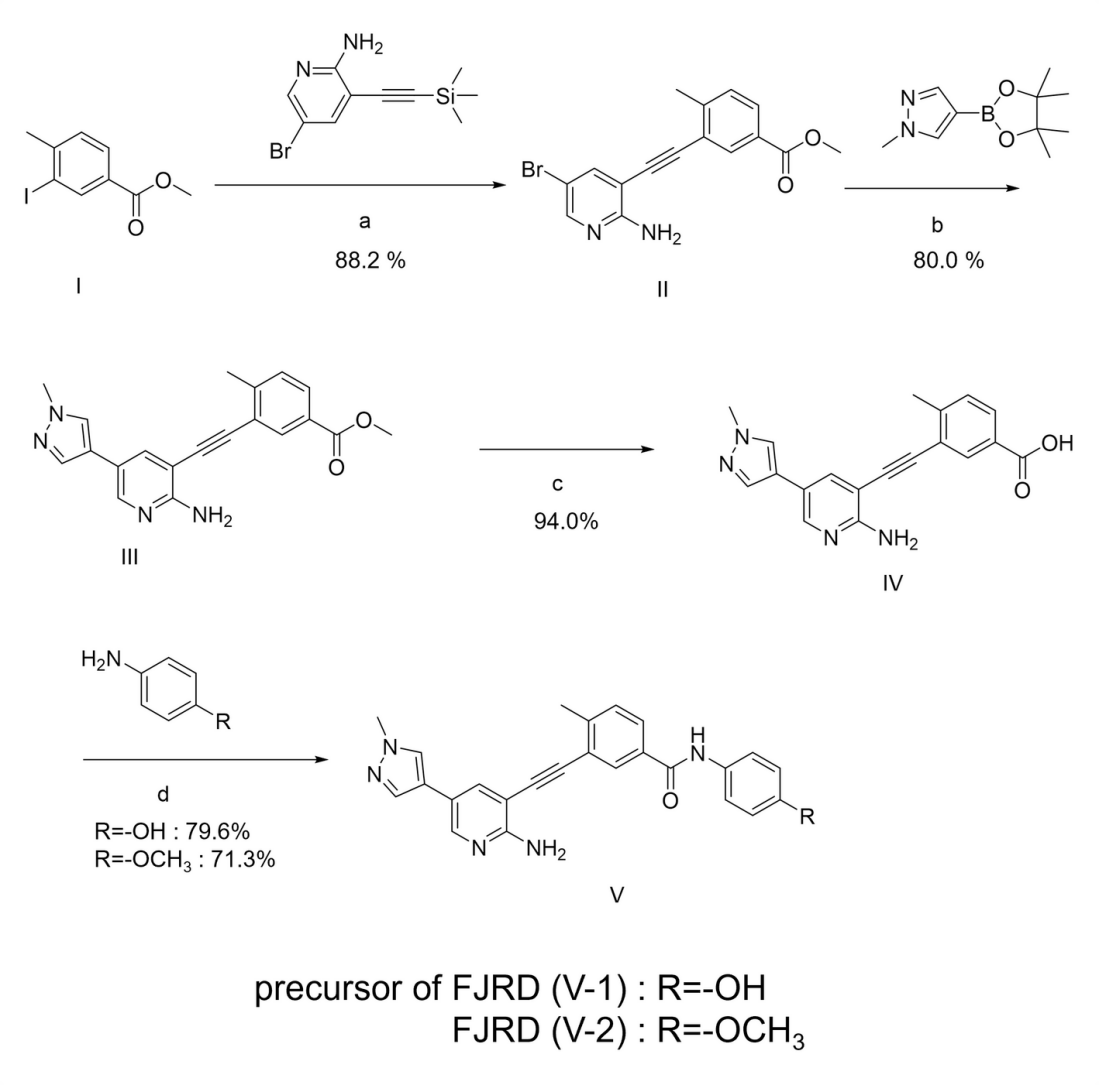
Chemical synthesis route of V-2 (FJRD) and V-1 (precursor for radiolabeling): (**a**) Pd(PPh_3_)_2_Cl_2_, CuI, CsF, Et_3_N, 6 h, 88.2%; (**b**) Pd(OAc)_2_, X-phos, K_2_CO_3_, THF, 6 h, 80.0%; (**c**) LiOH·H_2_O, 12 h, 94.0%; (d) HATU, DIPEA, 4 h, 79.6%

The synthetic pathways leading to compound **V-2** (FJRD) and **V-1** (precursor for radiolabelling, 3-((2-amino-5-(1-methyl-1*H*-pyrazol-4-yl)pyridin-3-yl)ethynyl)-*N*-(4-hydroxyphenyl)-4-methylbenzamide) are depicted in Fig. 1. The synthetic sequence began with commercially available halogenated heteroaromatic compounds, which underwent a Sonogashira cross-coupling reaction with ethynyltrimethylsilane to yield intermediate **II** (methyl 3-((2-amino-5-bromopyridin-3-yl)ethynyl)-4-methylbenzoate). Intermediate **II** was then coupled with a boronic acid ester to produce the key intermediate **III** (methyl 3-((2-amino-5-(1-methyl-1*H*-pyrazol-4-yl)pyridin-3-yl)ethynyl)-4-methylbenzoate). The hydrolysis of the methyl ester in intermediate **III** resulted in intermediate **IV** (3-((2-amino-5-(1-methyl-1*H*-pyrazol-4-yl)pyridin-3-yl)ethynyl)-4-methylbenzoic acid), which was subsequently condensed with various substituted anilines to complete the synthesis.

**Fig. 2:**
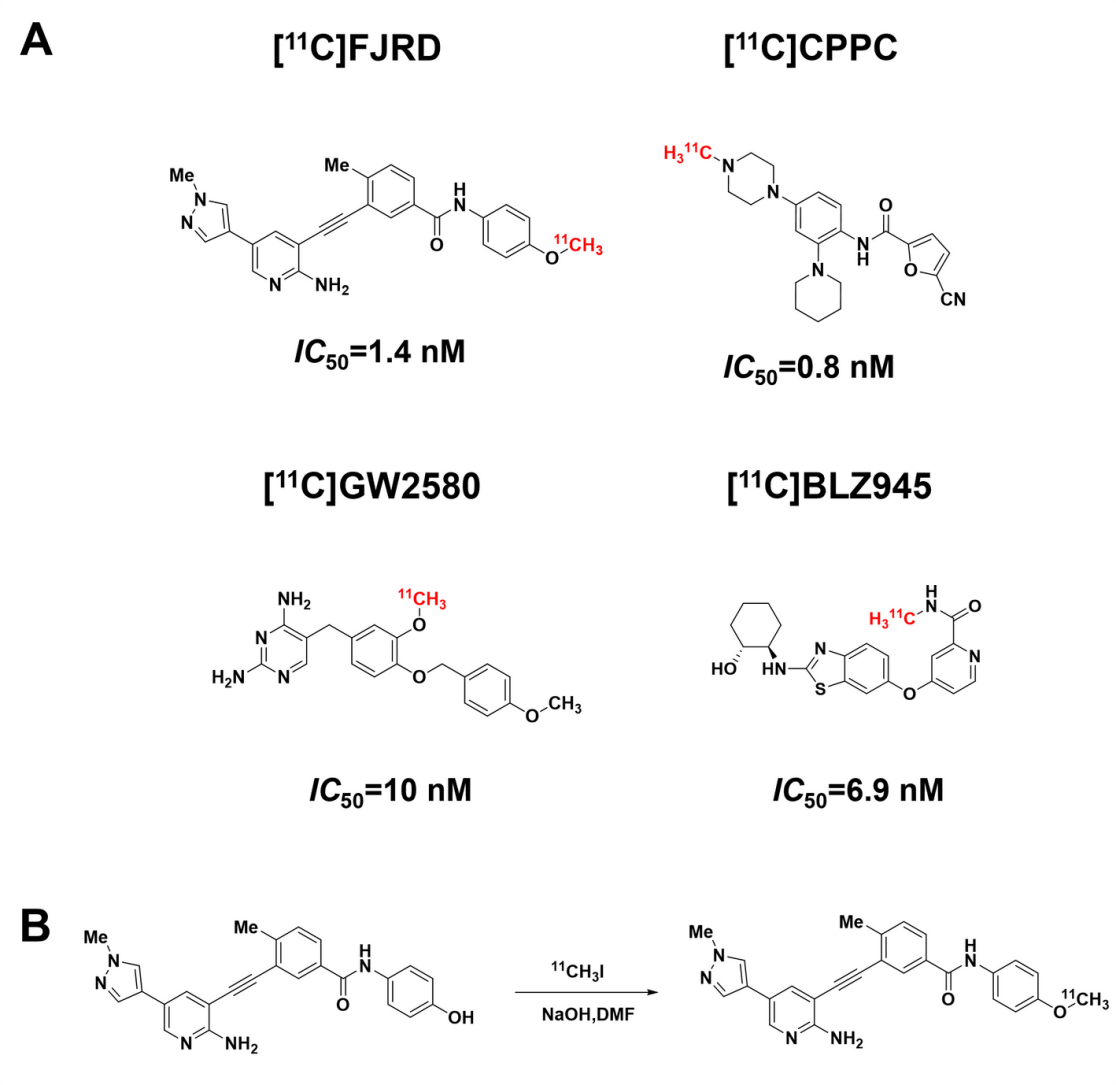
Chemical structures of CSF1R-PET tracers used in the present study and radiosynthesis of [^11^C]FJRD. (**A**) The affinities of [^11^C]FJRD (Xie et al. 2020) [^11^C]CPPC (Wildt et al. 2021a) [^11^C]GW2580 (Zhou at al. 2021) and [^11^C]BLZ945 (Wildt et al. 2021c) for CSF1R from the previous publications. (**B**) [^11^C]FJRD was radiosynthesized by one-step reaction of precursor and [^11^C]NaI

The chemical structures of three PET tracers used in the present study and radiolabeling reaction of [^11^C]FJRD were shown in Fig. 2 [^11^C]FJRD was radiosynthesized with a 36% radiochemical yield based on [^11^C]CO_2_ (decay-corrected to the end of irradiation), providing radioactivity and dependable quality for the evaluation experiments. The total synthesis time was averaged to be 45 min from the end of irradiation. The radiochemical purity of [^11^C]FJRD exceeded 90%, and the molar activity was approximately 120 GBq/µmol. The radiochemical purity of the final product solution was higher than 99% at the end of synthesis, with no significant impurity peaks observed in the high-performance liquid chromatography (HPLC) chromatogram. The radiochemical stability of [^11^C]FJRD was maintained for at least 90 min after the formulated product solution was stored at 25 °C, based on the evaluation of radiochemical purity using HPLC.

### In vivo whole body and brain imaging of [^11^C]FJRD in rodents

**Fig. 3:**
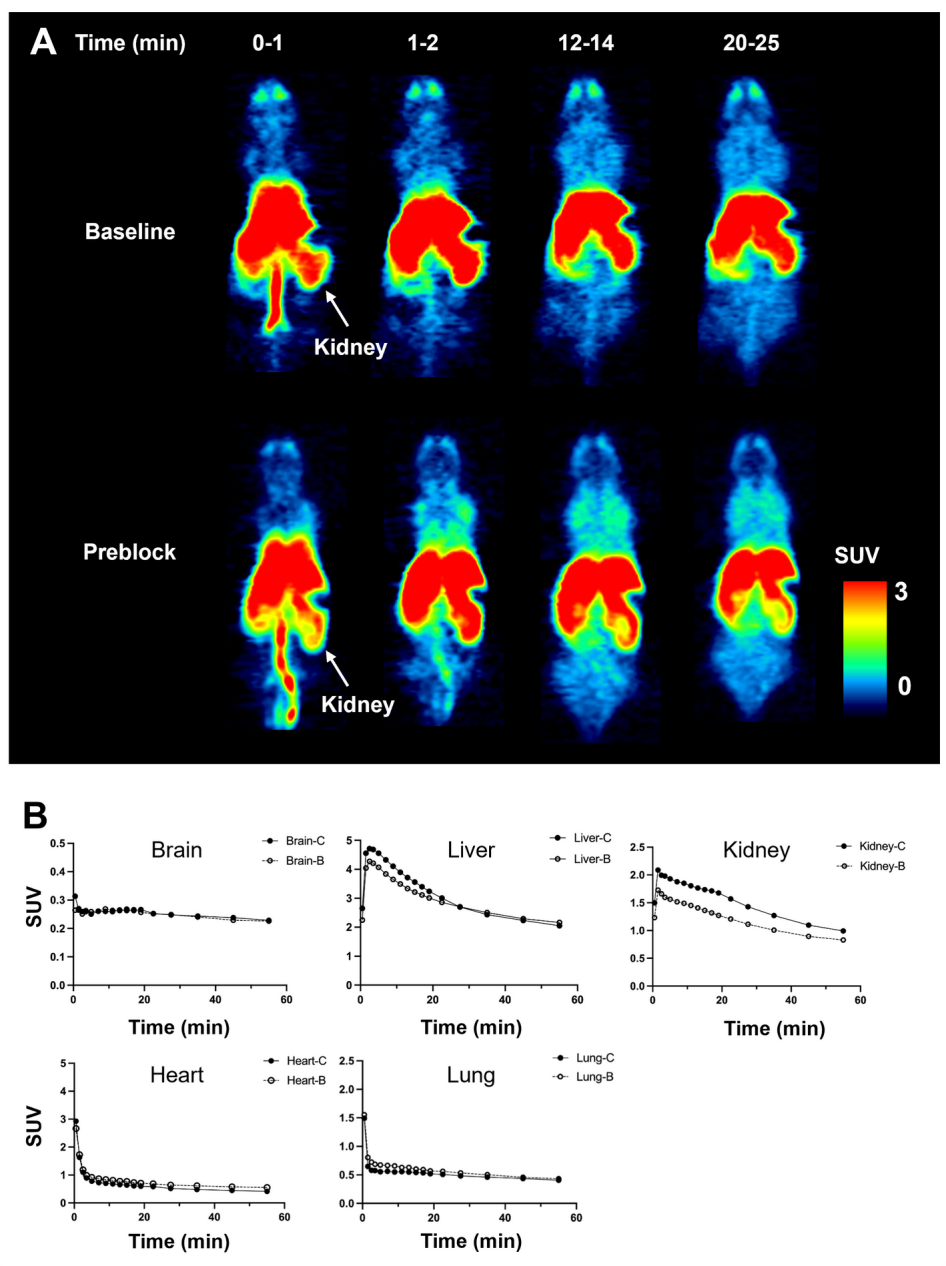
Whole body PET imaging with [^11^C]FJRD in mice. (**A**) Summation PET images of [^11^C]FJRD (0–1, 1–2, 12–14 and 20–25 min from left to right) with or without pretreatment of FJRD as indicated. (**B**) Time-activity curves in the brain, heart, lung, liver, and kidneys. Data are expressed as SUV. -C: Baseline; -B: Preblock

**Fig. 4:**
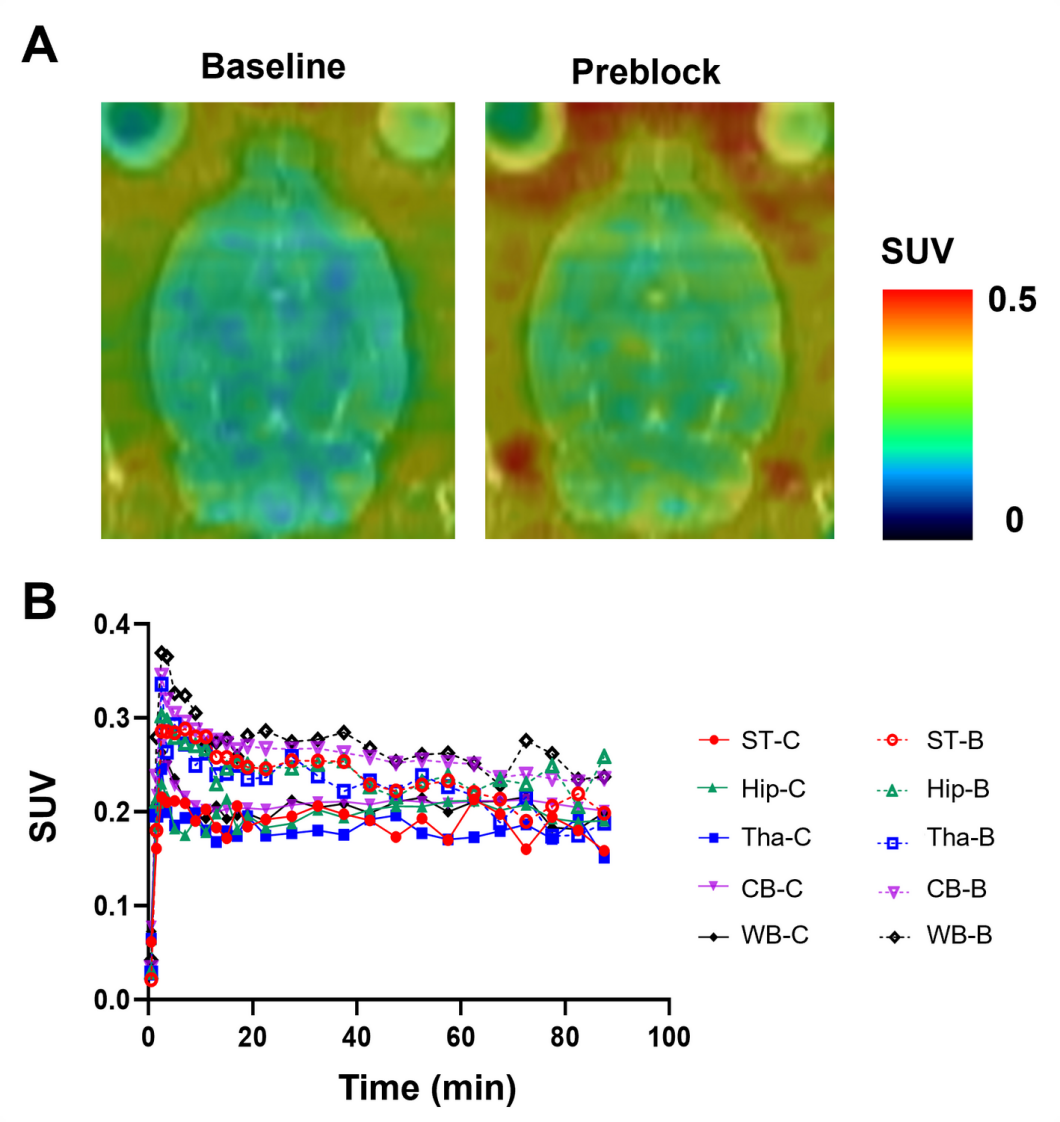
PET imaging of [^11^C]FJRD in rat brain. (**A**) Summation PET/MRI-fused images of [^11^C]FJRD (0–90 min) with or without pretreatment of FJRD. (**B**) Time-activity curves in the striatum (ST), hippocampus (Hip), thalamus (Tha), cerebellum (CB) and whole brain (WB). Data are expressed as SUV. -C: Baseline; -B: Preblock

PET images and time-activity curves showed rapid biodistribution of [^11^C]FJRD in various organs of normal mice, with standardized uptake value (SUV) peak of approximately 0.3-5 during the initial phase, followed by rapid washout, except in the brain. Cold blocking with unlabeled FJRD did not affect on its uptake in brain, heart, liver, lungs, but it decreased kidney uptake by approximately 25% (Fig. 3). PET imaging was also performed in normal rats to investigate uptake in brain subregions, including the striatum, hippocampus, thalamus, and cerebellum. As results, no overt difference in brain permeability or washout was detected among these brain subregions. Additionally, initial brain uptake of [^11^)C]FJRD in rats, with a peak SUV value of approximately 0.4, and washout were similar to those observed in mice. Pretreatment with FJRD slightly increased brain uptake, indicating no detectable specific binding (Fig. 4).

### In vitro autoradiography

**Fig. 5:**
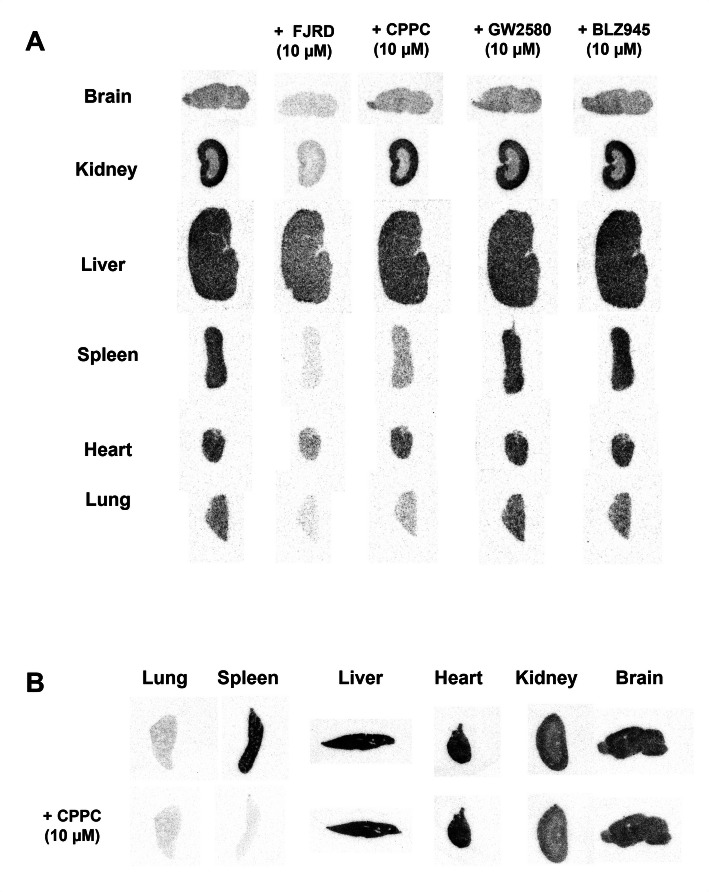
In vitro autoradiography with [^11^C]FJRD and [^11^C]CPPC in the peripheral and central organs of healthy mice. Representative in vitro autoradiographs of [^11^C]FJRD (**A**) and [^11^C]CPPC (**B**) in the various organs from healthy mice in the absence or presence of non-radioactive compounds including FJRD, CPPC, GW2580 and BLZ945 at the concentration of 10 µM as indicated

**Fig. 6:**
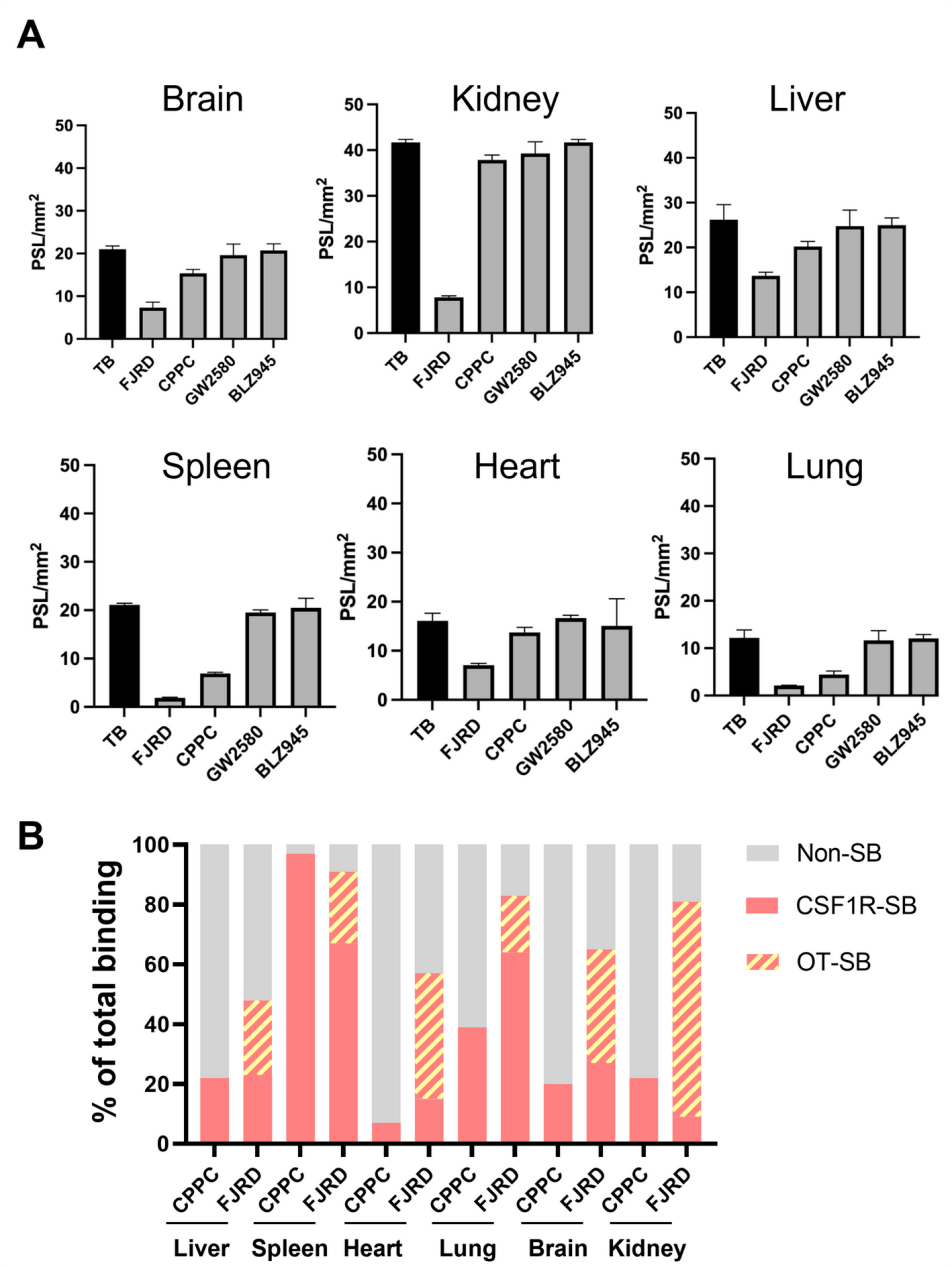
Quantitative analysis for in vitro binding properties of [^11^C]FJRD and [^11^C]CPPC. (**A**) Inhibitory effects of various CSF1R inhibitors on [^11^C]FJRD in vitro binding. Total binding (TB) is the binding of [^11^C]FJRD without the addition of any cold compound. *N* = 3 for each group. Data are expressed as mean ± SD. (**B**) The proportions of non-specific binding (non-SB), specific binding to CSF1R (CSF1R-SB) and specific binding to off-target (OT-SB) of [^11^C]FJRD (FJRD) and [^11^C]CPPC (CPPC) with respective total binding as 100%. Data from Fig. 5

In vitro autoradiograms of [^11^C]FJRD showed that decreased radioactivities by the addition of non-radioactive FJRD and CPPC were 91% vs. 67% in the spleen, 83% vs. 64% in the lungs, 81% vs. 9% in the kidneys, 65% vs. 27% in the brain, 56% vs. 15% in the heart and 48% vs. 23% in the liver, respectively (Figs. 5 and 6A). Meanwhile, only faint decreases in radioactivity were detected in all tested organs when either GW2580 or BLZ945 was added (Figs. 5A and 6A).

**Fig. 7:**
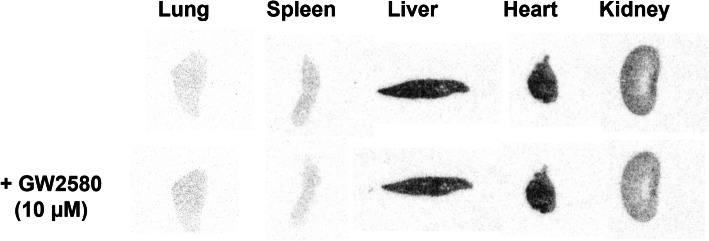
Representative in vitro autoradiography with [^11^C]GW2580 in the peripheral and central organs of healthy mouse in the absence or presence of non-radioactive GW2580 (10 µM)

Given that CPPC is a well-known high-affinity compound for CSF1R, reduced radioactivity by the addition of FJRD and CPPC are considered whole specific binding and specific binding to CSF1R (CSF1R-SB), respectively. The gap of two binding values represents specific binding to off-target (OT-SB). The proportions of CSF1R-SB, OT-SB and non-specific binding (non-SB) for [^11^C]CPPC and [^11^C]FJRD, with total binding set at 100%, are shown in Fig. 6B. The CSF1R-SB of [^11^C]CPPC and [^11^C]FJRD were highest in the spleen, with values of approximately 97% and 67%, respectively. The percentages of CSF1R-SB for [^11^C]FJRD are higher in the heart, lung and brain, compared to [^11^C]CPPC, suggesting its potential for more sensitive CSF1R-detectivity in these organs. However, unignorable amounts of OT-SB (ranging from 19 to 72%) are also abserved in the tested organs (Fig. 6B). In vitro autoradiography with [^11^C]GW2580 demonstrated that the addition of GW2580 did not decrease the binding of [^11^C]GW2580 in any of the organs tested, suggesting the absence of detectable specific binding under normal conditions (Fig. 7).

**Fig. 8:**
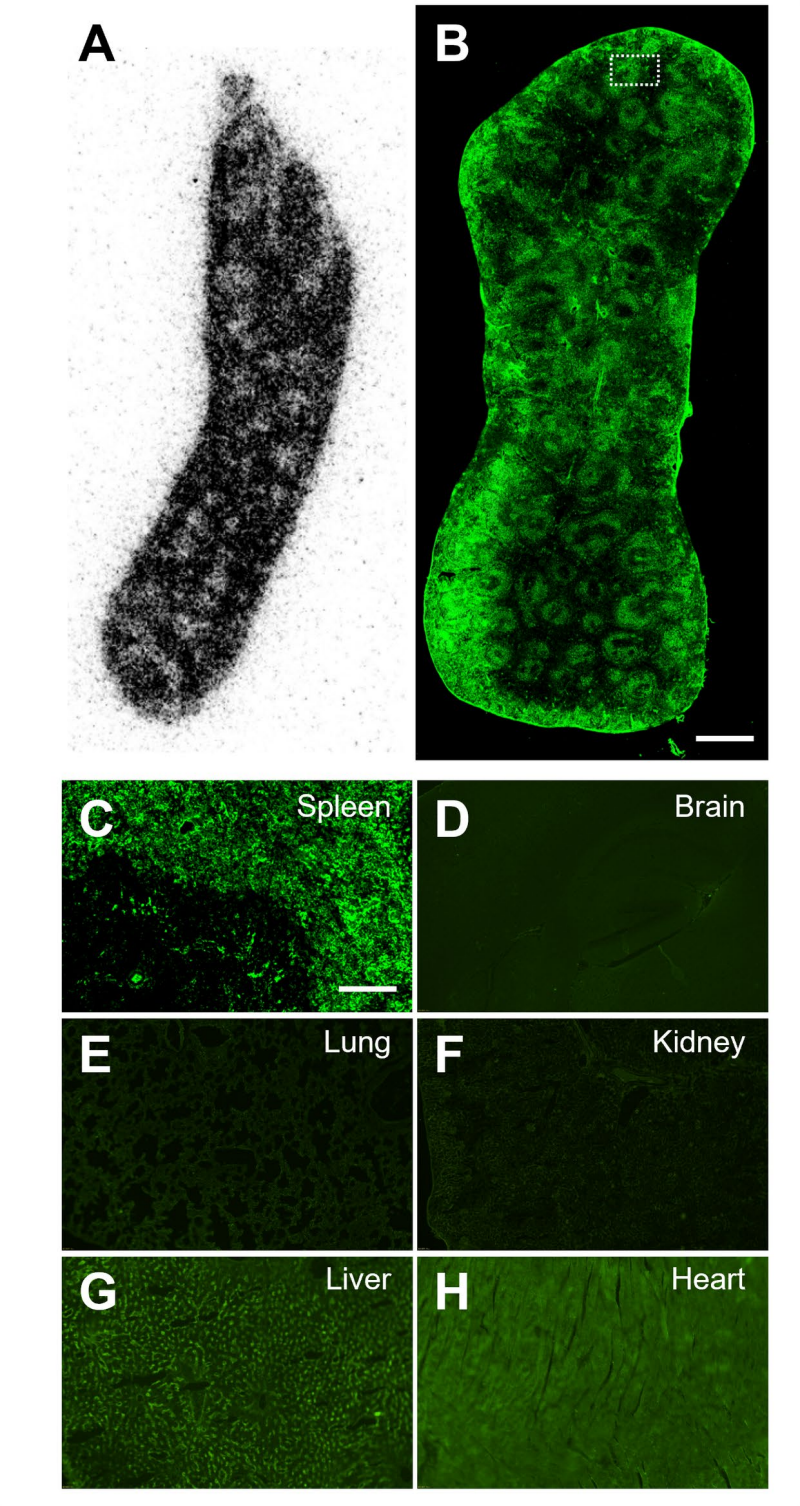
Immunohistochemstry for CSF1R in the normal mouse organs. In vitro autoradiographic image with [^11^C]CPPC (**A**) and immunohistochemistry images of CSF1R (B-H) in the tissue sections of the spleen (**A**-**C**), brain (**D**), lung (**E**), kidney (**F**), liver (**G**) and heart (**H**) from a three-month-old male C57BL/6J mouse. The area enclosed by dotted line in low-power image (**B**) was shown in high-power image (**C**) of the spleen. Scale bar: 1 mm in panel B; 100 μm in panel **C**-**H**

The immunostaining of CSF1R demonstrated abundant immunofluorescent signals with a mottled shape in the spleen, consistent with the spatial distribution of in vitro binding of [^11^C]CPPC in the autoradiographic image (Fig. 8A-C). No overt immuofluorescence was observed in the brain, lung, kidney or heart, except for a weak fluorescent signal in the liver (Fig. 8D-H). These findings support the autoradiography-based results, showing that CSF1R expression is significantly higher in the spleen compared to the brain, lung, kidney, heart and liver under normal conditions.

## Discussion

The present study involved the radiosynthesis of a novel *o*‑aminopyridyl alkynyl derivative [^11^C]FJRD, and the evaluation of its in vivo and in vitro binding properties in normal rodents. FJRD exhibits low-middle brain uptake in both mice and rats with a peak SUV value of approximately 0.5 (Figs. 3 and 4). The physicochemical properties of FJRD with a ClogD7.4 of 3.73, a MW of 436 g/mol, a PSA of 92.31 Å², and an HBD of 3, as calculated by ChemDraw, nearly meet general physicochemical parameters of compound for good BBB permeability (ClogD7.4 = 2–5, MW < 500 g/mol, PSA < 90 Å², and HBD < 3) (Hitchcock 2006). The present study has not conducted in vitro or in vivo assay to investigate the role of efflux transporters such as P-gp or BCRP in brain permeability of FJRD. We speculate that FJRD is not a strong substrate of brain efflux transporters, since the peak value of brain SUV of typical brain efflux transporter substrate is usually lower than 0.1 (Wildt et al. 2021a).

There are great amount of specific binding clearly detected in the investigated peripheral and central organs. However, the addition of three other CSF1R inhibitors (CPPC, GW2580 and BLZ945) only resulted in partial or no blocking effects on [^11^C]FJRD binding, suggesting that a substantial proportion of the binding is due to off-target binding to unknown molecules in these organs (Figs. 6 and 7). These findings provide a rationale to pay great attention to specificity for CSF1R as use radioactive *o*‑aminopyridyl alkynyl derivative such as [^11^F]4 for CSF1R imaging (An et al. 2023). There are several types of CSF1R-PET tracers with different core chemical structures. Except for the compounds used in the present study [^11^C]5 (3-(2-amino-5-(1-methyl-1*H*-pyrazol-4-yl)pyridin-3-yl)ethynyl)-*N*-(4-(2-fluoroethoxy)phenyl)-4-methylbenzamide) showed high-level specific binding in the normal rodent brain under in vitro conditions. However, its low brain permeability greatly limits its application for neuroinflammation imaging in CNS disorders (Wildt et al. 2021a). The lack of available data on its specific binding to peripheral organs also leaves its potential as a CSF1R imaging tracer for peripheral inflammation unclear. The present study also confirmed overt specific binding of CPPC, which was only detected in the spleen, consistent with a previous publication (Knight et al. 2021). A previous study has shown that CPPC exhibits submicomolar *IC*_50_ values for three kinases, including the insulin receptor (0.070 µM), tyrosine protein kinase LCK (0.022 µM), and vascular endothelial growth factor receptor 2 (0.074 µM), which raises concerns about potential off-target binding to these molecules (Knight et al. 2021). However, given the disparity between the faint specific binding observed in most organs and the abundant expression of these molecules in these tissues, such as the high expression level of vascular endothelial growth factor receptor 2 in the brain (Harris et al. 2018) [^11^C]CPPC might not bind to these molecules with high-performance. Furthermore, the spatial and shape consistency between in vitro binding and the abundant expression of CSF1R in the spleen (Fig. 8) strongly suggests that the observed specific bindig of [^11^C]CPPC in the spleen is indeed due to CSF1R. The partial blocking effect of CPPC on [^11^C]FJRD binding strongly indicates that these two compounds share a binding site on the CSF1R molecule, which is different with that for GW2580 or BLZ945. Given that BLZ945 showed great blockage for [^11^C]5 under in vitro conditions, there are at least two binding sites on CSF1R protein for these CSF1R imaging tracers. Berend et al. have reported a brain-permeable [^11^C]4 with a high affinity for CSF1R (*IC*_*50*_ = 12nM) and a similar core structure to GW2580. Its brain uptake was significantly decreased by pretreatment with the cold compound (Wildt et al. 2022). It is the only CSF1R PET tracer to date that demonstrates detectable specific binding in the living, healthy mouse brain. However, the lack of data on the in vitro binding of [^11^C]4 is disturbing, especially considering that [^11^C]GW2580 does not reveal specific binding in all major organs under in vitro autoradiographic conditions. Further investigation is required for its binding property.

Some protein molecules, such as VEGFR1, PDGFR-α, RET and c-Kit, may contribute to off-target binding of [^11^C]FJRD, since an analogue of FJRD inhibites these proteins by 23.3–70.4% at a 10 nM concentration (Wildt et al. 2022). SYHA1813, a dual-target inhibitor of VEGFR and CSF1R, also shares the core structure with FJRD and reveals very high affinity for VEGF receptors (VEGFRs), including VEGFR1, VEGFR2 and VEGFR3 with *IC*_*50*_ values of 2.8, 0.3 and 4.3 nM, respectively (Kang et al. 2023). These results imply VEGFRs as possible protein molecules providing the binding sites for [^11^C]FJRD. In the renal glomeruli, VEGFRs are highly expressed by both podocytes and glomerular endothelial cells (Schrijvers et al. 2004), which is consistent with the present finding of high off-target binding in kidneys (Fig. 6B).

Although off-target binding remains a major concern, the present study provides several key insights for CSF1R tracer development in the future. (1) FJRD and CPPC share a binding site that is abundant in the spleen, providing a positive-control material. (2) [^11^C]FJRD exhibits higher in vitro CSF1R-SB in heart, lung and brain compared to [^11^C]CPPC (Fig. 6B), suggesting its potential for more sensitive detection of CSF1R in these organs.

## Conclusions

In the present study, we developed a novel ^11^C-labeled *o*-aminopyridyl alkynyl derivative [^11^C]FJRD, as a potential CSF1R-PET tracer. Compared to the currently available tracers [^11^C]CPPC and [^11^C]GW2580, [^11^C]FJRD demonstrated enhanced sensitivity for detecting CSF1R in certain rodent organs. However, its modest brain-permeability and unignorable off-target binding highlight the need for further optimization to improve brain permeability and specificity for CSF1R.

## Methods

### General

All chemicals and organic solvents were purchased from Bidepharm and Energy Chemical, and used as supplied. ^1^H NMR spectroscopy and mass spectrometry (MS) were used to characterize the isolated compounds. NMR spectra were recorded on either a Qone AS400 instrument 400 MHz or a Bruker Avance III, 400 MHz instrument. All ^1^H NMR experiments were reported in units, parts per million (ppm), and measured relative to the signals of residual chloroform (7.28 ppm) or dimethyl sulfoxide (2.51 ppm) in the deuterated solvent, unless otherwise stated. The purities of the synthesized compounds were > 98%, as determined by analytical HPLC. Radio-HPLC was performed using a JASCO HPLC system (JASCO, Tokyo, Japan): effluent radioactivity was monitored using a NaI (Tl) scintillation detector system. Unless otherwise stated, radioactivity was measured using an IGC-3R Curiemeter (Hitachi Aloka Medical, Tokyo, Japan).

## The chemical synthesis of FJRD and its precursor for radiolabeling

The synthesis of compound FJRD follows previously reported routes with slight modifications (Xie et al. 2020). The chemical synthesis of intermediates is described as follow:

methyl 3-((2-amino-5-bromopyridin-3-yl)ethynyl)-4-methylbenzoate (**II**).

Under an argon atmosphere, a mixture of methyl 3-iodo-4-methylbenzoate (276 mg, 1 mmol), 5-bromo-3-((trimethylsilyl)ethynyl)pyridin-2-amine (270 mg, 1 mmol), Pd(PPh_3_)_2_Cl_2_ (35 mg, 0.05 mmol), CuI (19 mg,0.1 mmol), CsF (380 mg, 2.5 mmol), and Et_3_N (304 mg, 3 mmol) in MeCN (8.5 mL) was stirred at room temperature for 6 h. After the reaction completed, the reaction mixture was concentrated *in vacuo*. The obtained residue was purified by column chromatography on silica gel (heptane/ethyl acetate = 8/1, v/v) to afford **II** as a yellow solid (303 mg, 88.2%). LC-MS, single peak, m/e, 346.2 (M + 1).

methyl 3-((2-amino-5-(1-methyl-1*H*-pyrazol-4-yl)pyridin-3-yl)ethynyl)-4-methylbenzoate (**III**).

Under an argon atmosphere, a reaction vial was charged with **II** (270 mg, 0.8 mmol), methyl-4-(4,4,5,5-tetramethyl-1,3,2-dioxaborolan-2-yl)-1*H*-pyrazole (252 mg, 1.2 mmol), Pd(OAc)_2_ (18 mg, 0.08 mmol), X-phos (76 mg, 0.16 mmol), and K_2_CO_3_ (276 mg, 2.0 mmol). A mixture of tetrahydrofuran (THF, 5.5 mL) and H_2_O (1.4 mL) was then added to the vial. The mixture was stirred at 90 °C for 6 h. After the reaction completed, the reaction mixture was concentrated *in vacuo*. The reactant was purified by silica gel column chromatography (eluent: heptane/ethyl acetate = 2/1, v/v) to afford **III** as a yellow solid (221.5 mg, 80%). LC-MS, single peak, m/e, 347.1 (M + 1).

3-((2-amino-5-(1-methyl-1*H*-pyrazol-4-yl)pyridin-3-yl)ethynyl)-4-methylbenzoic acid (**IV**).

Compound **III** (200 mg, 0.58 mmol), and LiOH·H_2_O (61 mg, 1.44 mmol) were dissolved in a mixture of THF (5.5 mL), MeOH (0.5 mL), and H_2_O (1.4 mL). The reaction mixture was stirred at room temperature for 12 h. Upon completion of the reaction, the mixture was cooled to 0 °C, and the pH was adjusted to 4–5 by using 1 M HCl, leading to the formation of a crystalline solid. The mixture was stirred for an additional 30 min, and the precipitate was collected by filtration. The obtained solid was washed three times with water and dried to afford **IV** as a yellow solid (181 mg, 94% yield). LC-MS, single peak, m/e, 333.1 (M + 1).

3-((2-amino-5-(1-methyl-1*H*-pyrazol-4-yl)pyridin-3-yl)ethynyl)-*N*-(4-hydroxyphenyl)-4-methylbenzamide (**V-1**, precursor for radiolabeling).

A mixture of compound **IV** (175 mg, 0.53 mmol), 2-(7-azabenzotriazol-1-yl)-*N*,* N*,*N’N’*-tetramethyluronium hexafluorophosphate (HATU, 262 mg, 0.69 mmol), and DIPEA (206 mg, 1.59 mmol) in *N*,* N*-dimethylformamide (DMF, 5 mL) was stirred at room temperature for 30 min, followed by the addition of *p*-toluidine (72 mg, 0.66 mmol). The reaction was allowed to proceed at room temperature for 4 h. Upon completion, water was added to dilute the mixture, which was then extracted with ethyl acetate. The organic layer was collected, washed with a saturated saline solution, dried over Na_2_SO_4_, and concentrated *in vacuo*. The crude product was purified by silica gel chromatography (eluent: 100% ethyl acetate) to afford compound **V-1** as a yellow solid (184 mg, 79.6% yield). H NMR (400 MHz, DMSO*-d6*) δ 10.11 (s, 1 H), 9.32 (s, 1 H), 8.33 (d, *J* = 2.4 Hz, 1 H), 8.28 (d, *J* = 1.8 Hz, 1 H), 8.14 (s, 1 H), 7.91 (td, *J* = 3.6, 1.9 Hz, 2 H), 7.88 (d, *J* = 0.7 Hz, 1 H), 7.63–7.57 (m, 2 H), 7.52 (d, *J* = 8.1 Hz, 1 H), 6.83–6.78 (m, 2 H), 6.35 (s, 2 H), 3.90 (s, 3 H), 2.62 (s, 3 H). LC-MS, single peak, m/e, 438.2 (M + 1).

3-((2-amino-5-(1-methyl-1*H*-pyrazol-4-yl)pyridin-3-yl)ethynyl)-*N*-(4-methoxyphenyl)-4-methylbenzamide (**V-2**, FJRD).

A mixture of compound **IV** (200 mg, 0.6 mmol), HATU (267 mg, 0.78 mmol) and DIPEA (233 mg,1.8 mmol) in 5 mL DMF was stirred at room temperature for 30 min, followed by the addition of 4-aminophenol (71.4 mg, 0.58 mmol). The reaction was allowed to proceed at room temperature for 4 h. Upon completion, water was added to dilute the mixture, which was then extracted with ethyl acetate. The organic layer was collected, washed with a saturated saline solution, dried over Na_2_SO_4_, and concentrated *in vacuo*. The crude product was purified by silica gel chromatography (eluent: 100% ethyl acetate) to afford **V-2** (FJRD) as a yellow solid (181 mg, 71.3%). H NMR (400 MHz, DMSO*-d6*) δ 10.22 (s, 1 H), 8.33 (d, *J* = 2.4 Hz, 1 H), 8.30 (d, *J* = 1.8 Hz, 1 H), 8.14 (s, 1 H), 7.92 (dd, *J* = 9.6, 2.1 Hz, 2 H), 7.88 (d, *J* = 0.7 Hz, 1 H), 7.77–7.71 (m, 2 H), 7.53 (d, *J* = 8.1 Hz, 1 H), 7.03–6.96 (m, 2 H), 6.35 (s, 2 H), 3.90 (s, 3 H), 3.81 (s, 3 H), 2.62 (s, 3 H). LC-MS, single peak, m/e, 424.1 (M + 1).

### Radiochemistry

Cyclotron-produced [^11^C]CO_2_ was introduced into 0.4 M LiAlH_4_ in anhydrous THF (0.3 mL). After the THF was evaporated, the remaining complex was reacted with 57% hydroiodic acid (0.3 mL) to yield [^11^C]CH_3_I. The [^11^C]CH_3_I was distilled under heating and transferred under a stream of N_2_ gas to a solution containing the precursor (1 mg) and NaOH (5 µL, 0.5 M) in DMF (0.3 mL) at -15 °C. After the trapping process was completed, the reaction mixture was heated to 80 °C for 5 min. HPLC separation was conducted using a Capcell PAK UG80 C18 column (10 × 250 mm; Shiseido, Osaka, Japan) with a mobile phase of MeCN/H_2_O/Et_3_N (5/5/0.001, v/v/v) at a flow rate of 5.0 mL/min. The radioactive fraction corresponding to [^11^C]FJRD (t_R_ = 9.2 min) was collected in a flask pre-loaded with Tween 80 (0.075 mL) and ethanol (0.3 mL). The collected fraction was then evaporated to dryness, redissolved in 3 mL of sterile normal saline containing 3.3% (v/v) Tween 80 and 0.8% (v/v) ascorbic acid, and filtered through a 0.22 μm Millipore filter (Billerica, MA, USA). The identity of [^11^C]FJRD was confirmed by co-injection with unlabeled FJRD on a reverse-phase analytical HPLC using a Capcell PAK UG80 C18 column (4.6 × 250 mm) with a mobile phase of MeCN/H_2_O/Et_3_N (55/45/0.001, v/v/v) at a flow rate of 1.0 mL/min (t_R_ = 6.4 min). Samples of formulated [^11^C]FJRD with known radioactivity (~ 6 MBq) were applied into HPLC and the chemical amounts of FJRD in the injectates were measured for absorbance at 254 nm by standard curves, which were generated under the same HPLC conditions. Molar activities were expressed as GBq per µmol.

### Experimental animals

Three-month-old male C57Bl/6J mice and ten-week-old male Sprague-Dawley (SD) rats were procured from CLEA-Japan (Tokyo, Japan). Upon arrival, the animals were housed in the vivarium facilities at the National Institute of Radiological Sciences, where they were kept under standard laboratory conditions with controlled temperature and humidity. Food and water were provided ad libitum to ensure their well-being. The animals were acclimatized to the facility environment before the commencement of any experimental procedures, adhering to best practices in animal care and research protocols.

### In vitro autoradiographic analysis

In vitro autoradiography was conducted following a previously published protocol with minor modifications (Ji et al. n vit^2008^). Organ sections were first pre-incubated in PBS for 30 min to hydrate the tissue. Following this, the sections were incubated at room temperature for 30 min with incubation solution (3.8% BSA in PBS) containing radioactive tracer (5 nM [^11^C]FJRD or [^11^C]CPPC or [^11^C]GW2580) in the presence or absence of unlabeled compounds (10 µM), as indicated. After incubation, the sections were washed twice with PBS, each for 2 min, to remove unbound radioligands. The sections were then briefly immersed in distilled water for 10 s to remove any remaining salts. The tissues were subsequently air-dried under gentle warmth and then affixed to an imaging plate (BAS-MS2025; GE Healthcare). Exposure times on the imaging plate were optimized based on the specific requirements of each experiment. Radiolabeling was then detected by scanning the imaging plate using the BAS-5000 system (FUJIFILM, Tokyo, Japan), allowing for precise autoradiographic analysis of the radiotracer distribution within the sections. Regions of interest (ROIs) were carefully placed on whole organ. Total and non-specific binding were expressed by radioactivity in the absence and presence of unlabeled compounds, respectively. Specific binding was calculated as total binding minus non-specific binding.

### Small-animal PET imaging

Mice or rats were anesthetized with 1.5-2.0% isoflurane and carefully positioned on the pre-heated scanner bed of small-animal PET scanners (Focus 220 for mice and Inveon for rats; Siemens Medical Solutions Knoxville, TN, USA), as previously described (Ji et al. 2008). Following positioning, mice and rats were intravenously injected with a solution of [^11^C]FJRD (37–55 MBq for mice and 48–66 MBq for rats), with a carrier dose of FJRD (15.5–23.0 nmol/kg for mice and 1.3–1.9 nmol/kg for rats), immediately followed by a PET scan. For preblock, the animals were intravenously injected with a solution of FJRD (1 mg/kg body weight) 5 min prior to the administration of [^11^C]FJRD. Dynamic PET data were continuously collected over 60 min for whole-body mouse scans and 90 min for brain scans in rats. The energy window for detecting ^11^C emissions was set between 350 and 750 keV. Decay correction factors were applied, calculated from the start of the acquisition to a reference time point, defined as the initiation of the first acquisition in the first animal. Image reconstruction was performed using a maximum-a-posteriori algorithm to generate single-frame average images for qualitative analysis, and filtered backprojection with a 0.5-mm Hanning filter for generating dynamic images used in quantitative assessments. Volumes of interest (VOIs) were delineated on brain regions of rats and peripheral organs of mice as indicated in the corresponding figures, utilizing PMOD image analysis software (PMOD Technologies Ltd, Zurich, Switzerland).

### Immunohistochemistry

The normal mouse organs (spleen, brain, lung, kidney, liver and heart) were fixed with 4% paraformaldehyde, cryoprotected using 20% sucrose in phosphate buffer, and then sectioned into 10-µm-thick frozen sections using a cryostat. Immunohistochemistry was performed according to a standard protocol, with a mouse monoclonal anti-CSF1R antibody (1:500; SANTA CRUZ, sc-46662) as the primary antibody and a fluorophore-conjugated secondary antibody (1:500; goat anti-mouse IgG; Abcam) as described in our previous publication (Zhou et al. 2021). Immunohistochemical images were captured by a fluorescence microscope/digital camera (BZ-X700, Keyence).

## Data Availability

Data can be obtained upon request.

## Abbreviations

CSF1R: Colony stimulating factor 1 receptor
PET: Positron emission computed tomography
SUV: Standardized uptake value
%ID/g: Percentage of injection dose per gram tissue
X-phos: 2-Dicyclohexylphosphino-2’,4’,6’-triisopropylbiphenyl
THF: Tetrahydrofuran
DIPEA: *N*, *N*-Diisopropylethylamine
HATU: 2-(7-Azabenzotriazol-1-yl)-*N*, *N*,*N*’*,N*’-tetramethyluronium hexafluorophosphate
